# Pre-Treatment T2-WI Based Radiomics Features for Prediction of Locally Advanced Rectal Cancer Non-Response to Neoadjuvant Chemoradiotherapy: A Preliminary Study

**DOI:** 10.3390/cancers12071894

**Published:** 2020-07-14

**Authors:** Bianca Petresc, Andrei Lebovici, Cosmin Caraiani, Diana Sorina Feier, Florin Graur, Mircea Marian Buruian

**Affiliations:** 1 Department of Radiology, “George Emil Palade” University of Medicine, Pharmacy, Science and Technology of Târgu Mureș, 540139 Târgu Mureș, Romania; petresc.bianca@stud19.umfst.ro (B.P.); mircea.buruian@umfst.ro (M.M.B.); 2 Department of Radiology, Emergency Clinical County Hospital Cluj-Napoca, 400006 Cluj-Napoca, Romania; diana.feier@umfcluj.ro; 3 Department of Radiology, “Iuliu Hațieganu” University of Medicine and Pharmacy Cluj-Napoca, 400012 Cluj-Napoca, Romania; 4 Department of Medical Imaging, “Iuliu Hațieganu” University of Medicine and Pharmacy Cluj-Napoca, 400012 Cluj-Napoca, Romania; 5 Department of Radiology, Regional Institute of Gastroenterology and Hepatology “Prof. Dr. Octavian Fodor”, 400158 Cluj-Napoca, Romania; 6 Department of Surgery, “Iuliu Hațieganu” University of Medicine and Pharmacy Cluj-Napoca, 400012 Cluj-Napoca, Romania; florin.graur@umfcluj.ro; 7 Department of Surgery, Regional Institute of Gastroenterology and Hepatology “Prof. Dr. Octavian Fodor”, 400158 Cluj-Napoca, Romania; 8 Department of Radiology, Emergency Clinical County Hospital Târgu Mureș, 540136 Târgu Mureș, Romania

**Keywords:** locally advanced rectal cancer, radiomics, neo-adjuvant chemoradiotherapy, non-responders, magnetic resonance imaging

## Abstract

Locally advanced rectal cancer (LARC) response to neoadjuvant chemoradiotherapy (nCRT) is very heterogeneous and up to 30% of patients are considered non-responders, presenting no tumor regression after nCRT. This study aimed to determine the ability of pre-treatment T2-weighted based radiomics features to predict LARC non-responders. A total of 67 LARC patients who underwent a pre-treatment MRI followed by nCRT and total mesorectal excision were assigned into training (*n* = 44) and validation (*n* = 23) groups. In both datasets, the patients were categorized according to the Ryan tumor regression grade (TRG) system into non-responders (TRG = 3) and responders (TRG 1 and 2). We extracted 960 radiomic features/patient from pre-treatment T2-weighted images. After a three-step feature selection process, including LASSO regression analysis, we built a radiomics score with seven radiomics features. This score was significantly higher among non-responders in both training and validation sets (*p* < 0.001 and *p* = 0.03) and it showed good predictive performance for LARC non-response, achieving an area under the curve (AUC) = 0.94 (95% CI: 0.82–0.99) in the training set and AUC = 0.80 (95% CI: 0.58–0.94) in the validation group. The multivariate analysis identified the radiomics score as an independent predictor for the tumor non-response (OR = 6.52, 95% CI: 1.87–22.72). Our results indicate that MRI radiomics features could be considered as potential imaging biomarkers for early prediction of LARC non-response to neoadjuvant treatment.

## 1. Introduction

Colorectal cancer is the third most common cancer worldwide and the second leading cause of oncologic-related mortality around the globe [1]. Rectal cancers account for approximately 30% of the total cases of colorectal malignancies [2]. Currently, the standard-of-care treatment for patients with locally advanced rectal cancer (LARC) is neoadjuvant chemotherapy (nCRT) followed by total mesorectal excision (TME) [3,4,5]. However, the individual response to nCRT is very heterogeneous, ranging from a pathological complete response to no tumor regression or even disease progression [6,7,8,9,10]. The percentage of patients who do not achieve tumor regression after nCRT, defined as non-responders (NR), is reported to be between 7 and 30% [11,12,13,14,15]. Additionally, the potential side effects of nCRT can be very serious such as hematologic, gastrointestinal or dermatologic effects, incontinence or sexual dysfunction [15,16,17,18,19] and 14–27% of patients with LARC who received this regimen developed acute or long-term grade 3–4 toxic effects as reported by Sauer et al. [20]. It is therefore argued that for non-responders the side effects of nCRT may outweigh its benefits, while nCRT does not improve the clinical outcome of these patients [21,22,23]. Early prediction of NR before the beginning of neoadjuvant therapy could be of great value in order to avoid ineffective treatment and to develop a more tailored strategy of care such as a primary surgical intervention or an intensified treatment regimen.

The rectal cancer response to nCRT can be assessed by means of endorectal ultrasound or MRI examination performed at the end of the neoadjuvant treatment [24,25]. However, the final confirmation of the tumor response can only be made by histopathologic examination of the surgical specimens and identifying non-responders before surgery still remains a great challenge. Extensive efforts have been made in various fields, focusing on gene expression, mutations and molecular metabolites as potential noninvasive biomarkers for predicting the response to nCRT in LARC patients [26,27,28,29,30].

In the radiology field, a new method, radiomics, has been developed based on the idea that medical images contain more information than what the human eye can perceive [31]. Radiomics represents a non-invasive, high-throughput post-processing technique, which extracts large amounts of quantitative features from routinely acquired medical images [32,33]. By measuring the distribution and relationships of gray levels within a lesion, radiomics texture features can reveal nonvisual information associated with tumor heterogeneity and the microenvironment; thus providing a detailed and comprehensive characterization of the tumor phenotype [34,35,36]. The heterogeneity of a tumor can be potentially related with its response to treatment and prognosis since heterogeneous tumors are prone to have a more aggressive behavior and increased resistance to treatment [37,38,39]. Since they are high dimensional inputs, radiomics features have been employed in multiple machine learning and deep learning algorithms for developing radiomics signatures useful in the oncology field [40,41,42,43]. Recent studies have demonstrated the efficacy of radiomics features as biomarkers for lesion characterization, therapy guidance and tumor prognosis among various types of cancers, including rectal cancer [44,45,46,47,48].

Based on these previous promising results, we hypothesize that magnetic resonance (MR)-based radiomics features may have a potential role in predicting locally advanced rectal cancer resistance to nCRT. The aim of the present study was to investigate the value of T2 weighted-based radiomics features extracted from baseline MRI for the prediction of non-responding rectal tumors and to develop a radiomics score based on these parameters.

## 2. Results

### 2.1. Patients Characteristics

A total of 67 patients (mean age: 60.36 ± 10.89) were included in this study. The 67 patients were split into two groups: training group (44 patients) and validation group (23 patients). In the training group, 17 patients were classified according to the tumor regression grade (TRG) as non-responders (NR) having TRG = 3, while the remaining 27 were classified as responders (R), 5 of them having TRG = 1 and 22 with TRG = 2. In the validation group, 8 patients were identified as non-responders, while 15 subjects were added to the responders group (5 with TRG 1 and 10 with TRG 2).

Patients and tumor characteristics are summarized in Table 1. No significant differences were observed in terms of age, gender, T stage and N stage between responders and non-responders in both training and validation groups. In the training group, tumor length, tumor differentiation grade and mesorectal fascia involvement (MRF) were significantly different among non-responders versus responders, however, this was not confirmed in the validation dataset.

### 2.2. Feature Selection and Radiomics Score Construction—Training Set

A total of 960 radiomics features were extracted from T2W images for each patient. After the inter-reader agreement evaluation, only features with an intraclass coefficient ≥ 0.75 were included in the further steps (874 features). To develop the radiomics signature, we first performed a univariate analysis of radiomics features between the responders and non-responders groups. 74 features with an adjusted *p* value < 0.05 were included in the next step (Appendix Table A1).

**Figure 1:**
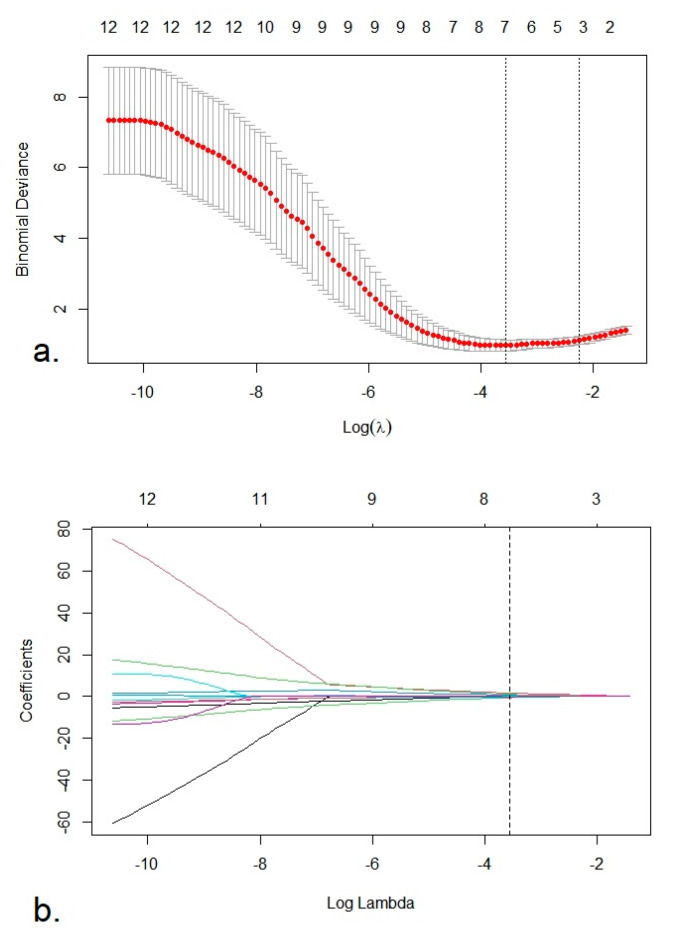
(**a**) Selection of the tuning parameter lambda (λ) using 10-fold cross validation. Binomial deviances from the least absolute shrinkage and selection operator (LASSO) regression cross-validation model were plotted as a function of log (λ). The dotted vertical lines were drawn at the optimal λ value based on the minimum criteria and 1 standard error of the minimum criteria. The optimal λ value of 0.028 and log (λ) = −3.57 was selected. (**b**). LASSO coefficient profiles of the 12 radiomics features. The vertical dotted line was plotted at the optimal λ value, resulting in seven radiomics feature with non-zero coefficients.

**Figure A1:**
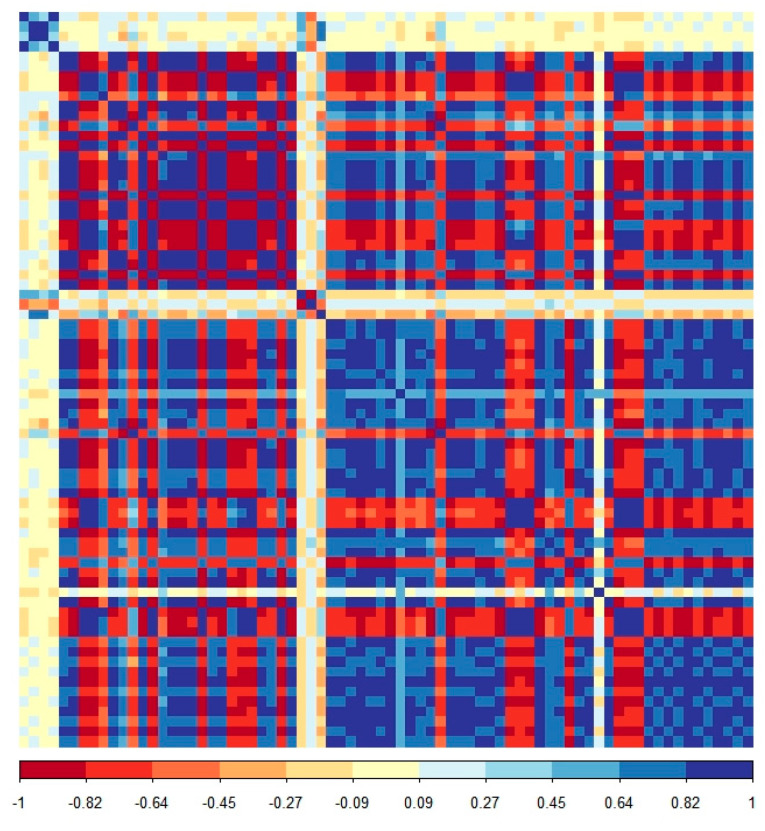
Correlation matrix of the radiomics features using Spearman coefficients of correlation.

These features were secondly reduced to 12 potential predictors by applying a Spearman correlation analysis, excluding redundant features with correlation coefficients >0.9/<−0.9. The correlation matrix is shown in Appendix Figure A1. Finally, through a least absolute shrinkage and selection operator (LASSO) binary logistic regression method using 10-fold cross-validation, seven radiomics features with non-zero coefficients were selected to construct the radiomics score (Rad-Score). The feature selection process using the LASSO algorithm is shown in Figure 1a,b.

The radiomics score was a linear combination of these 7 features, weighted according to their respective LASSO coefficients (presented in Table 2). The equation for calculating the Rad-Score is the following:

$$\mathrm{Rad}-\mathrm{Score}=\sum_{i=0}^{7}C_{i}*X_{i}+b$$

where *C_i_* is the coefficient of the *i*th feature, *X_i_* the *i*th feature and *b* the intercept.

**Figure 2:**
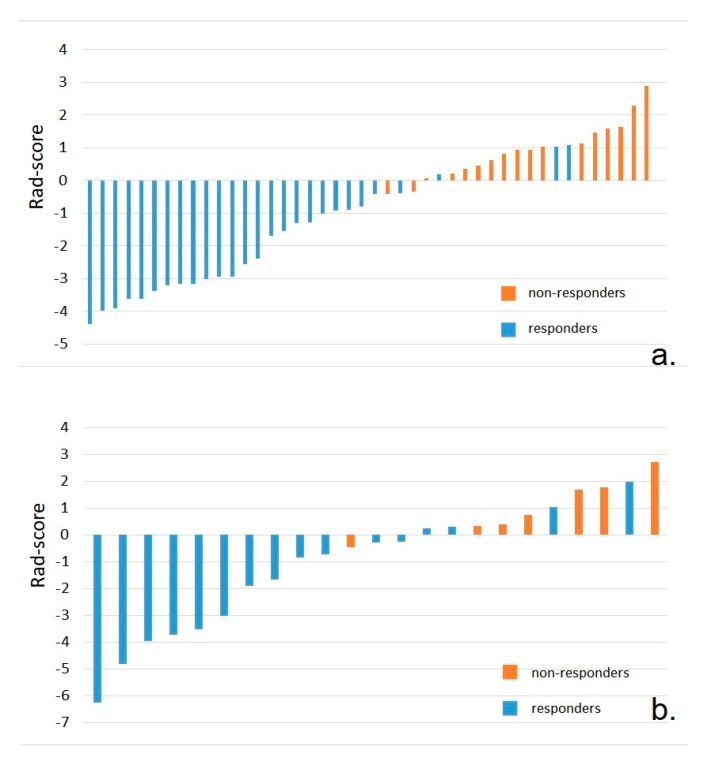
(**a**). Bar charts of the radiomics signature for each patient in the training set. (**b**). Bar charts of the radiomics signature for each patient in the validation set. The orange bars indicate the patients in the non-responders group, while the blue bars represent patients in the responders group.

The radiomic score was calculated for each patient (Figure 2a). There was a significant difference of the Rad-Score between non-responders and responders, patients from the first group having higher values (0.92 ± 0.87 vs. −2.00 ± 1.55, *p* < 0.001).

### 2.3. Performance of the Radiomics Score—Training Set

**Figure 3:**
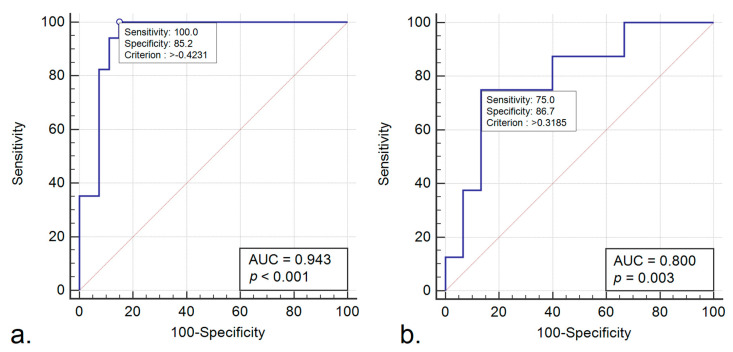
(**a**). Receiver operating characteristic (ROC) curve of the radiomics score for predicting locally advanced rectal cancer (LARC) non-responders—training set. (**b**). ROC curve of the radiomics score for predicting LARC non-responders—validation set.

Figure 3a shows the receiver operating characteristic (ROC) curve for the radiomics score in the training set. The radiomics score predicted a rectal cancer non-response to nCRT with an area under the curve (AUC) of 0.94 (95% CI, 0.82–0.99) and accuracy of 91%, resulting in a sensitivity of 100% (95% CI, 80.5–100%) and a specificity of 85.2% (95% CI, 66.3–95.8%) for the cut-off value of −0.42. Additionally, the radiomics signature achieved a positive predictive value (PPV) of 81% (95% CI, 58.1–94.6%) and a negative predictive value (NPV) of 100% (95% CI, 85.2–100%).

ROC analysis was also performed for each of the seven radiomics features to evaluate their individual diagnostic performance. Table 3 shows the AUC, sensitivity, sensibility, PPV and NPV for the determined cut-off values. However, the performance of the Rad-Score was higher than the performance of each separate feature for distinguishing non-responders.

**Figure 4:**
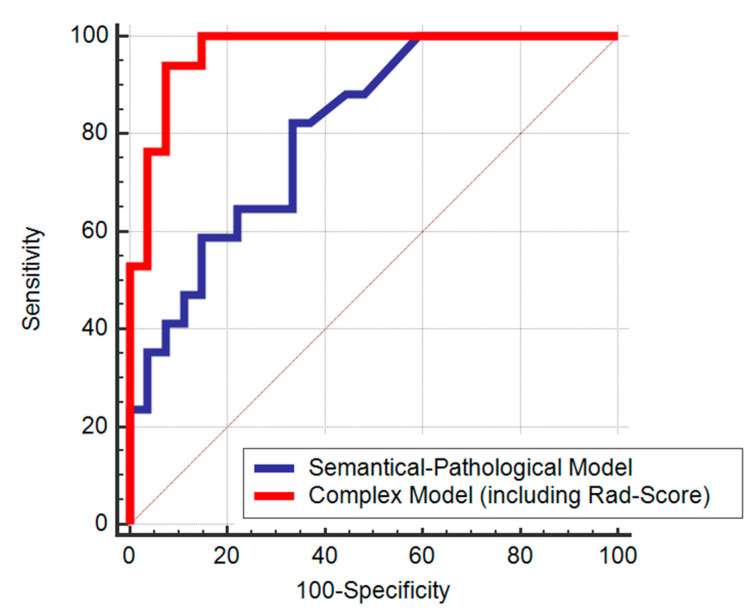
ROC curves of the semantical-pathological and complex models score for predicting LARC non-responders.

Using the variables with significant difference among responders and non-responders in the training group: tumor length, tumor differentiation grade and MRF status, we conducted a multivariate logistic regression analysis to develop a semantical-pathological model for the prediction of LARC non-response (Table 4). Afterwards, we constructed a complex model, adding the radiomics score to the semantical-pathological model (Table 5). In the complex model, the Rad-Score was identified as an independent predictor of the LARC lack of response to neoadjuvant treatment (OR = 6.52, CI: 1.87–22.72). The ROC curves of the semantic-pathological model and the complex model are shown in Figure 4. Adding the radiomics score to the first model improved its performance for the differentiation of non-responders (AUC = 0.97 (95% CI, 0.66–0.91) vs. AUC = 0.80 (95% CI, 0.87–0.99), *p* = 0.007).

### 2.4. Validation of the Radiomics Score

In the validation set, the Rad-Scores of non-responders patients were significantly higher than the scores of responders (0.52 ± 1.74 vs. −1.62 ± 2.36, *p* = 0.03; Figure 2b). The performance of the Rad-Score for discrimination of non-responders was confirmed in the validation set, yielding an AUC = 0.80 (95% CI, 0.58–0.94) (Figure 3b). For the previous cut-off value of −0.42 (established in the training group), in the validation group there were recorded sensitivity = 75% (95% CI, 34.9–96.8%), specificity = 60% (95% CI, 32.3–83.7%), PPV = 50% (95% CI, 21.1–78.9%), NPV = 81.8% (95% CI, 48.2–97.7%) and accuracy of 65%.

## 3. Discussion

Our study evaluated the ability of radiomic features extracted from T2-weighted images to help differentiate non-responders (NR) from responders. In recent years, there was an increased interest in the field of radiomics for predicting the rectal cancer response to nCRT, with numerous research being conducted in this respect. The majority of studies focused on predicting the pathological complete response, using a single MRI sequence (T2-WI or apparent diffusion coefficient (ADC) maps) or a multiparametric approach [49,50,51,52,53,54,55]. Additionally, there are several studies that investigated the performance of radiomic features to discriminate good responders [56,57,58,59,60]. In contrast to our research, these investigations either used other pathologic classifications for the quantification of tumor regression grade such as Dworak [57] or Mandard [59] or the authors divided their study population different from our approach, including in the non-responders’ group both patients with a TRG score of 2 and 3 [56,58,60]. In our research, we used the Ryan TRG classification and we considered patients with a TRG score of 3 as non-responders, while patients with TRG 2 were included in the responders’ group. We chose this split method since TRG 3 is equivalent to significant fibrosis outgrown by cancer/no fibrosis with extensive residual cancer, meaning that the neoadjuvant treatment had little to no effect on the rectal tumor, while TRG 2 represents residual cancer outgrown by fibrosis, indicating that the rectal tumor tissue achieved a moderate degree of regression after nCRT [61]. To the best of our knowledge, there are only a few published papers that studied the performance of radiomics features to predict the resistance of rectal cancer to nCRT [62,63,64] and all of them applied the same criteria of defining the rectal cancer non-response as in this present paper. However, two of them used only parameters extracted from the ADC maps for the development of the prediction model [62,63]. In the study by Zhou et al., the authors built a multiparametric prediction model, including features from T2 weighted images, diffusion weighted images and contrast-enhanced T1-weighted images [64].

As well as these previous studies, we analyzed only the pre-treatment, baseline MRI scans. For the prediction of non-responders, it is important to identify these patients in an early phase, before or shortly after the beginning of nCRT in order to be able to adjust their regimen or to offer them alternative treatment options. However, contrary to the above-mentioned investigations, we selected only T2-WI images for our radiomic analysis and model construction. High-resolution T2-weighted sequence is the most important one for the assessment and local staging of rectal cancer since it offers a good visualization of rectal wall layers and it provides good contrast between tumor, surrounding fat and mesorectal fascia [65]. Therefore, it is an essential sequence included in the standard rectal MR protocol and it is always performed when evaluating rectal tumors [66]. Moreover, when compared to images obtained by another sequence, T2-WI images have better stability in appearance. With respect to diffusion weighted images, these can be affected by distortion or by susceptibility artifacts, which may alter tumor segmentation and feature extraction [67,68]. The contrast-enhanced T1-WI sequence is not routinely included in the MRI protocol for rectal cancer staging. The potential of radiomics features extracted from MRI T2-weighted images for predicting a pathological complete response of rectal cancer was demonstrated in several recent studies, which reported promising results of their radiomics models with AUCs ranging from 0.69 to 0.93 [51,52,57,69,70,71]. In contrast to MRI, a recent study had demonstrated that radiomics features extracted from CT images showed no predictive power for complete pathological response in LARC [72], while another research showed that MRI T2-WI radiomics model performed better than CT radiomics model for predicting the LARC response to nCRT [73].

In addition to the previous papers that investigated the performance of radiomics for the prediction of non-response in LARC, our radiomics analysis was performed using the whole tumor volume rather than using only a single section. Although segmenting a volume of interest (VOI) is a time-consuming process, we believe that selection of a single slice might not be very representative, and the information obtained from a 3D lesion might be more reliable for the characterization of the entire tumor.

Regarding the image preprocessing and feature extraction, we extracted radiomics features acquired from both unfiltered and filtered images, applying Laplacian of Gaussian (LoG) filters and Wavelet filters. The majority of the features included in our final radiomic score were obtained from filtered images using the wavelet filters. Wavelet filters are useful for signal denoising and there are several radiomics studies that applied them for different purposes [74,75,76], including in the field of rectal cancer [72,77,78]. In a recent research of He et al., which aimed to develop an MRI-based radiomics signature for tumor grading of rectal carcinoma, the most relevant features included in their classifier were derived from wavelet-filtered images [77]. Additionally, in the predictive model constructed by Liang et al. for the prediction of metachronous liver metastasis in patients with rectal cancer, their optimal selected feature model based on T2-WI consisted only of parameters extracted from filtered images using wavelet filters [78]. Additionally, wavelet filters have proved to be useful even for preprocessing CT images, a significant proportion of the selected features from the recent study of Hamerla et al. for the differentiation of complete LARC responders being obtained from wavelet-filtered images [72].

Our radiomics model mainly consisted of first-order features derived from histogram and second order features derived from the grey-level co-occurrence matrix (GLCM). First-order features describe the distribution of voxel intensities within a region/volume of interest, without considering spatial interactions [34]. Second order or textural features that characterize the spatial relationship between voxels are calculated from different matrices. GLCM quantifies the frequency of specific gray values along a distance or direction [79].

As for the predictive performance of our constructed radiomics model, it achieved AUCs of 0.94 and 0.80 in the training and validation datasets, both indicators showing a good performance. Our results are in concordance with Zhou et al., their radiomics score yielding an AUC of 0.822 [64]. However, their model was a multiparametric one and it included only one feature from T2-WI [64]. In their research, Zhou et al. also developed a single-modality, T2-WI based radiomics signature, which achieved an AUC of 0.602 and 0.630 in the training and validation cohorts, both values being lower than the AUC obtained in the present paper. One possible explanation for these discrepant results can be the different pre-processing and feature extraction algorithm, our study applying in addition wavelet filters, while Zhou et al. extracted the radiomics features only from original and LoG-filtered images. Moreover, our observations are similar with the findings of Liu et al. and Yang et al., which reported AUCs of 0.908 and 0.83 respectively for predicting resistant rectal adenocarcinoma using ADC maps as a basis for feature extraction [62,63]. Additionally, our study was relatively in line with the prior investigations of Yi et al. and Shi et al., whose radiomics models achieved AUCs of 0.90 and 0.91 respectively for distinguishing good responders from non-good responders, although their classification of non-responders was slightly different than ours [57,58]. In our research, the multivariate analysis indicated that the radiomics score was the only independent predictor for the differentiation of LARC non-responders, having an odds ratio of 6.52.

Although our results were significant, this study had some limitations. First, this was a single-institution, retrospective study with a small sample size of patients. Our statistical approach included only one classification method: the binary logistic regression method and more advanced classifiers may provide better prediction performance. Therefore, further larger studies, preferably prospective and multicentric, are needed to overcome these limitations and to validate the reported data in order to provide a better generalization and to assess the potential for clinical translation of our proposed radiomics signature.

## 4. Materials and Methods

### 4.1. Study Population

The Institutional Review Board of Regional Institute of Gastroenterology and Hepatology “Prof. Dr Octavian Fodor” Cluj-Napoca approved this retrospective, HIPAA (Health Insurance Portability and Accountability Act)-compliant study and waived the requirement for written informed consent (IRB 5337/22.04.2020). We performed a retrospective analysis in our electronic medical database for patients diagnosed with rectal cancer who underwent an MR examination for initial tumor staging between January 2017 and May 2019. The inclusion criteria were patients diagnosed with locally advanced rectal cancer on pre-treatment (baseline) MR examination who underwent long-course neoadjuvant CRT followed by total mesorectal excision. The exclusion criteria were as follows: patients who did not complete the standard nCRT (12 patients), patients who did not undergo surgical resection (5 patients), patients without available tumor regression grading information on the pathological record (6 patients) and MR examinations with insufficient quality for proper analysis (9 patients). Our final study population consisted of 67 eligible patients, who were divided into a training group (44 patients) and into a validation group (23 patients).

### 4.2. Image Acquisition

All patients underwent rectum MRI scans 1 or 2 weeks before the start of chemoradiation. The examinations were performed in a single institution, using a 1.5 Tesla MRI scanner (Symphony TIM upgrade, Siemens AG, Erlangen, Germany) with an 8-channel phased array body coil. The protocol included three T2 weighted turbo spin-echo (TSE) sequences in the sagittal, oblique-axial high-resolution and oblique-coronal high-resolution planes. DWI images were obtained in axial planes using EPI sequences at three b-values (b50, b400 and b 800 s/mm^2^) and restriction of diffusion was quantified by the ADC value. The parameters of the MRI sequences are provided in Table 6. No bowel preparation was received prior to the MRI examination.

### 4.3. Reference Standard

Surgically resected specimens were histopathologically examined and tumor regression grading (TRG) was established according to the criteria proposed by Ryan et al. [80]: TRG 1 = no viable cancer cells, or single cells, or small groups of cancer cells; TRG 2 = residual cancer outgrown by fibrosis and TRG 3 = significant fibrosis outgrown by cancer, or no fibrosis with extensive residual cancer.

### 4.4. Preprocessing, Segmentation and Feature Extraction

**Figure 5:**
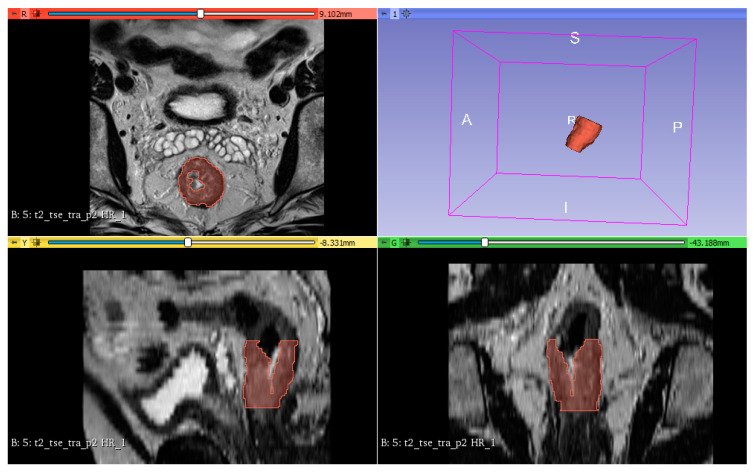
An example of rectal tumor VOI segmentation.

All oblique-axial high-resolution T2-WI TSE image were retrieved from a picture archiving and communication system (PACS, Carestream, Canada) for image segmentation. Three radiologists: one radiology resident (Bianca Petresc) and two senior radiologists with 10 and 8 years of experience (Cosmin Caraiani and Andrei Lebovici) in gastrointestinal MRI reviewed all images and reached a consensus about the tumor location. Afterwards, the radiology resident and one senior radiologist (C.C.) have independently segmented the whole tumor volume, by manually delineating the lesion on each consecutive slide, excluding the uninvaded rectal wall and the intestinal lumen. The segmentations were then independently reviewed by the other senior radiologist (Andrei Lebovici) and adjustments were made by agreement when necessary. All the three radiologists were blinded to the pathological results. The 3D segmentation of the tumors was performed using a designated, open source software 3D Slicer, version 4.10.2 (available at: https://www.slicer.org/). Figure 5 shows an example of tumor segmentation.

Prior to radiomic feature extraction, all MR images were pre-processed by the method proposed by van Griethuysen et al. [81] for noise reduction, intensity normalization and discretization. Six categories of radiomics features were extracted: 14 shape features, 18 first-order features, 22 gray level co-occurrence matrix (GLCM) features, 16 gray-level size zone matrix (GLSZM) features, 16 gray-level run length matrix (GLRLM) features and 14 gray-level dependence matrix (GLDM) features. Features were automatically extracted from images with and without preprocessing filters. The filters included Laplacian of the Gaussian (LoG) filter with sigma values of 3.0 and 5.0 mm and wavelet filter, using either a low band-pass filter or a high-band pass filter in x, y, z directions. Finally, a total of 934 radiomic features were obtained. PyRadiomics version 2.1.2. [82] was used for pre-processing and feature extraction and detailed information about the PyRadiomics configuration is provided in Supplementary File S1.

### 4.5. Feature Selection and Statistical Analysis

The inter-reader agreement was evaluated using the intraclass coefficient (ICC) between the features extracted from the radiology resident’s segmentation and the senior radiologist’s segmentation. Only features with an ICC ≥ 0.75 were selected for further feature selection process, resulting in a total of 874 features. All radiomics extracted features were normalized by transforming the data into standardized ranges across all subjects with a mean of 0 and an SD of 1 (*z*-score transformation). To control overfitting in our radiomics model, we used three feature selection steps. First, we performed a univariate analysis using the Mann–Whitney *U*-test to identify the features with significant difference between the responders and non-responders groups. The Benjamini–Hochberg (BH) method was used to adjust for multiple testing. BH-adjusted *p* values less than 0.05 were considered significant. Secondly, the Spearman correlation analysis was used to reduce redundancy. This was conducted between any 2 features and when the Spearman coefficient was >0.9/< − 0.9 the feature with the higher *p*-value in the univariate analysis was eliminated. Finally, regularized multivariate logistic regression analysis with the least absolute shrinkage and selection operator (LASSO) conducted by a 10 cross-fold cross-validation was applied to the previously selected features. The final selected features were combined into a radiomics score, which was calculated by a linear combination of the selected features weighted by their respective LASSO coefficients. Receiver operating characteristic (ROC) curve analysis was conducted and area under the curve (AUC), sensitivity, specificity and accuracy were calculated to evaluate the performance of the radiomics score for the prediction of non-responders in both training and validation sets. Multivariate analysis using binary logistic regression (enter method) was performed to identify independent predictors of non-responders, including as independent variables the patients’ and tumors’ characteristics and the radiomics score. *p* values < 0.05 were considered statistically significant. All statistical analysis was performed using commercially available software SPSS Statistics for Windows, version 18.0 (SPSS Inc., Chicago, IL, USA) and free available R software version 3.6.3 using the “glmnet”, “selectiveinference” and “corrplot” packages.

## 5. Conclusions

Our study has shown that radiomics features extracted from pre-treatment T2-weighted images may play a role as a potential imaging biomarker to predict rectal cancer resistance to neoadjuvant CRT. However, to confirm its ability for the prediction of LARC non-response, our proposed radiomics model needs to be externally validated in larger, multicentric studies.

## Figures and Tables

**Table 1:** Baseline characteristics of the study population.

Variable	Training		*p* Value	Validation		*p* Value
	Responders (*n* = 27)	Non-Responders (*n* = 17)		Responders (*n* = 15)	Non-Responders (*n* = 8)	
Age (years)	58.33 ± 1.38	56.47 ± 2.94	0.572	66.40 ± 9.60	64.12 ± 16.10	0.722
Gender			0.185			0.057
Male	22 (66.7%)	11 (33.3%)		5 (45.5%)	6 (54.5%)	
Female	5 (45.5%)	6 (54.5%)		10 (83.3%)	2 (16.7%)	
Tumor length (cm)	58.81 ± 17.99	68.88 ± 11.60	0.04 *	62.06 ± 15.01	61.50 ± 13.89	0.929
Tumor differentiation grade			0.01 *			0.149
Well differentiated	15 (83.3%)	3 (16.7%)		5 (83.3%)	1 (16.7%)	
Moderately differentiated	11 (55.0%)	9 (45.0%)		9 (69.2%)	4 (30.8%)	
Poor differentiated	1 (16.7%)	5 (83.3%)		1 (25.0%)	3 (75.0%)	
Clinical tumor stage (cT)			0.907			0.779
T2	4 (66.7%)	2 (33.3%)		2 (50%)	2 (50%)	
T3	19 (59.4%)	13 (40.6%)		11 (68.8%)	5 (31.2%)	
T4	4 (66.7%)	2 (33.3%)		2 (66.7%)	1 (33.3%)	
Clinical nodal stage (cN)			0.3			0.757
N1	8 (72.7%)	3 (27.3%)		8 (66.7%)	4 (33.3%)	
N2	19 (57.6%)	14 (42.4%)		7 (63.6%)	4 (36.4%)	
MRF			0.024 *			0.679
Positive	4 (33.3%)	8 (66.7%)		10 (62.5%)	6 (37.5%)	
Negative	23 (71.9%)	9 (28.1%)		5 (71.4%)	2 (28.6%)	

* Statistically significant *p* < 0.05; results are presented as the mean ± standard deviation or number (%).

**Table 2:** List of selected radiomics features and their coefficients for calculation of the radiomics score.

Variable	Coefficient	95% CI	
		Upper	Lower
Intercept	−0.875		
log-sigma-5-0-mm-3D_glszm_SmallAreaEmphasis	1.621	−0.460	4.428
wavelet-LHL_glcm_Correlation	−0.581	−3.954	3.322
wavelet-LHL_firstorder_10Percentile	0.660	−1.536	16.268
wavelet-HHL_glcm_MCC	−0.074	−4.724	20.345
wavelet-HHL_glcm_Imc1	0.984	−0.232	8.356
wavelet-HHL_firstorder_Kurtosis	−0.144	−7.629	7.938
wavelet-HHL_glszm_SmallAreaHighGrayLevelEmphasis	−0.070	−4.027	24.241

**Table 3:** Individual diagnostic performance of the selected radiomics features.

Variable	Cut-Off Value	AUC	Accuracy (%)	Se (%)	Sp (%)	PPV (%)	NPV (%)
logsigma5_0mm_3D_glszm_SmallAreaEmphasis	−0.24	0.80	72.7	94.1	59.3	59.3	94.1
wavelet-LHL_glcm_Correlation	−0.58	0.74	65.6	100	44.4	53.1	100.0
wavelet_LHL_firstorder_10Percentile	−0.28	0.71	75.0	94.1	62.9	61.5	94.4
wavelet-HHL_glcm_MCC	0.34	0.69	63.6	88.2	48.1	51.7	86.7
wavelet_HHL_glcm_Imc1	0.01	0.75	75.0	88.2	66.7	62.5	90.0
wavelet_HHL_firstorder_Kurtosis	0.33	0.69	68.2	70.6	66.7	57.1	78.3
wavelet_HHL_glszm_SmallAreaHighGrayLevel Emphasis	0.13	0.71	68.2	82.3	59.3	56.0	84.2

**Table 4:** Multivariate logistic regression analysis for the prediction of the non-responders―semantical-pathological model.

Variable	Coefficient	Std. Error	*p* Value	Odds Ratio (OR)	95% CI	
					Upper	Lower
Tumor length	0.03	0.02	0.23	1.03	0.98	1.08
Tumor differentiation grade—poorly differentiated	−2.67	1.23	0.10	0.07	0.05	1.30
MRF status—positive	−1.38	0.84	0.30	0.25	0.06	0.77
Constant	0.906	2.14	0.67	2.47		

**Table 5:** Multivariate logistic regression analysis for the prediction of non-responders―complex model.

Variable	Coefficient	Std. Error	*p* Value	Odds Ratio (OR)	95% CI	
					Upper	Lower
Tumor length	0.008	0.06	0.889	1.0008	0.90	1.13
Tumor differentiation grade—poorly differentiated	−4.561	3.30	0.167	0.10	0.00	7.92
MRF status—positive	−0.904	1.52	0.551	0.40	0.02	7.92
Rad-Score	1.876	0.64	0.003 *	6.52	1.87	22.72
Constant	4.11	5.14	0.42	61.17		

* Statistically significant *p* < 0.05.

**Table 6:** MRI parameters.

MRI Parameter	TSE T2-Weighted Image			DWI
	Sagittal	HR Coronal Oblique	HR Axial Oblique	
TR (ms)	3500	3500	4000	5800
TE (ms)	91	91	80	96
Slice no	28	25	25	30
Bandwidth (Hz/pixel)	391	391	391	1132
FOV (mm)	220	220	200	250
Slice thickness (mm)	3	4	3	4
Matrix	256 × 256	256 × 256	256 × 256	136 × 160
Acquisition time (min)	4	5.5	6	4.5

## Supplementary Materials

The following are available online at https://www.mdpi.com/2072-6694/12/7/1894/s1, Supplementary File S1: PyRadiomics Configuration.

## Appendix A

**Table A1:** List of selected radiomics features by univariate analysis for classification of non-responders vs. responders.

Feature	*p* Value *
log-sigma-5-0-mm-3DglszmSizeZoneNonUniformityNormalized	0.001
log-sigma-5-0-mm-3Dglszm_SizeZoneNonUniformity	0.013
log-sigma-5-0-mm-3DglszmGrayLevelNonUniformity	0.044
log-sigma-5-0-mm-3Dglszm_SmallAreaEmphasis	0.001
wavelet-LHL_glcm_Contrast	0.035
wavelet-LHLglcmDifferenceEntropy	0.033
wavelet-LHLglcmInverseVariance	0.044
wavelet-LHLglcmIdm	0.044
wavelet-LHLglcmCorrelation	0.009
wavelet-LHLglcmSumEntropy	0.049
wavelet-LHLglcmImc2	0.033
wavelet-LHLglcmImc1	0.020
wavelet-LHLglcmDifferenceAverage	0.031
wavelet-LHLglcmId	0.042
wavelet-LHLgldmDependenceEntropy	0.033
wavelet-LHLgldmSmallDependenceEmphasis	0.042
wavelet-LHLgldmDependenceNonUniformityNormalized	0.044
wavelet-LHLfirstorderInterquartileRange	0.037
wavelet-LHLfirstorderUniformity	0.037
wavelet-LHLfirstorderRobustMeanAbsoluteDeviation	0.042
wavelet-LHLfirstorderEntropy	0.047
wavelet-LHLfirstorder10Percentile	0.020
wavelet-LHLglrlmGrayLevelNonUniformityNormalized	0.042
wavelet-LHLglrlmRunVariance	0.044
wavelet-LHLglrlmRunEntropy	0.047
wavelet-LHLglszmGrayLevelVariance	0.047
wavelet-LHLglszmGrayLevelNonUniformityNormalized	0.031
wavelet-LHLglszmZonePercentage	0.047
wavelet-LLHgldmDependenceEntropy	0.014
wavelet-LLHglszmGrayLevelNonUniformityNormalized	0.031
wavelet-LLHglszmSizeZoneNonUniformity	0.049
wavelet-HHLglcmJointAverage	0.039
wavelet-HHLglcmSumAverage	0.039
wavelet-HHLglcmContrast	0.042
wavelet-HHLglcmDifferenceEntropy	0.047
wavelet-HHLglcmDifferenceVariance	0.037
wavelet-HHLglcmAutocorrelation	0.037
wavelet-HHLglcmSumEntropy	0.049
wavelet-HHLglcmMCC	0.037
wavelet-HHLglcmSumSquares	0.039
wavelet-HHLglcmClusterProminence	0.021
wavelet-HHLglcmImc2	0.010
wavelet-HHLglcmImc1	0.005
wavelet-HHLglcmDifferenceAverage	0.047
wavelet-HHLglcmClusterTendency	0.033
wavelet-HHLgldmGrayLevelVariance	0.031
wavelet-HHLgldmHighGrayLevelEmphasis	0.023
wavelet-HHLgldmSmallDependenceHighGrayLevelEmphasis	0.029
wavelet-HHLgldmDependenceNonUniformityNormalized	0.047
wavelet-HHLgldmLargeDependenceEmphasis	0.042
wavelet-HHLgldmLargeDependenceLowGrayLevelEmphasis	0.039
wavelet-HHLgldmDependenceVariance	0.039
wavelet-HHLfirstorderMeanAbsoluteDeviation	0.049
wavelet-HHLfirstorderMaximum	0.049
wavelet-HHLfirstorderRootMeanSquared	0.035
wavelet-HHLfirstorderMinimum	0.029
wavelet-HHLfirstorderRange	0.033
wavelet-HHLfirstorderVariance	0.031
wavelet-HHLfirstorderKurtosis	0.039
wavelet-HHLglrlmGrayLevelVariance	0.031
wavelet-HHLglrlmGrayLevelNonUniformityNormalized	0.044
wavelet-HHLglrlmRunVariance	0.047
wavelet-HHLglrlmLongRunEmphasis	0.047
wavelet-HHLglrlmShortRunHighGrayLevelEmphasis	0.027
wavelet-HHLglrlmShortRunEmphasis	0.047
wavelet-HHLglrlmLongRunHighGrayLevelEmphasis	0.031
wavelet-HHLglrlmRunPercentage	0.047
wavelet-HHLglrlmRunEntropy	0.042
wavelet-HHLglrlmHighGrayLevelRunEmphasis	0.021
wavelet-HHLglrlmRunLengthNonUniformityNormalized	0.047
wavelet-HHLglszmGrayLevelVariance	0.026
wavelet-HHLglszmSmallAreaHighGrayLevelEmphasis	0.019
wavelet-HHLglszmZonePercentage	0.049
wavelet-HHLglszmHighGrayLevelZoneEmphasis	0.023

* Adjusted *p*-values for multiple tests (Benjamini–Hochberg method).
