# Supplementary material for: Pre-Treatment T2-WI Based Radiomics Features for Prediction of Locally Advanced Rectal Cancer Non-Response to Neoadjuvant Chemoradiotherapy: A Preliminary Study
Source: Cancers (Basel). 2020 Jul 14;12(7):1894. doi: 10.3390/cancers12071894 (PMC7409205; doi:10.3390/cancers12071894)
Supplement: Supplementary file 1 [file cancers-12-01894-s001.pdf]

cancers

Supplementary Files

Pre-Treatment T2-WI Based Radiomics Features for Prediction of Locally Advanced Rectal Cancer Non-Response to Neoadjuvant Chemoradiotherapy: A Preliminary Study

Bianca Petresc, Andrei Lebovici, Cosmin Caraiani, Diana Sorina Feier, Florin Graur and Mircea Marian Buruian

PyRadiomics Configuration

setting:

Image discretization:
The gray values were discretized using a fixed-bin width of 5
binWidth: 5

Normalization:
Images were normalized before feature extraction to 0 mean and 100 standard deviation
normalize: true
normalizeScale: 100

Resampling:
Images were interpolated to isotropic voxels with 2 mm sides using a B-Spline interpolator
interpolator: 'sitkBSpline'
resampledPixelSpacing: [2, 2, 2]

Resegmentation
Segmentations were resegmented by excluding voxels which differed > 3 sigma from the mean
resegmentRange: [-3,3]
resegmentMode: sigma

first order specific settings:
When normalizing, gray values below the mean will be negative. Shifting by 300 (3 StdDevs * 100) ensures that the majority of voxels is positive (only outliers >3 SD lower than the mean will be negative).
voxelArrayShift: 300

Misc:
default label value.
label: 1

imageType:

Selection of filters
Original: {}

```
LoG:
        sigma: [3.0, 5.0]
    Wavelet: {}

    featureClass:
      # Selection of the extracted radiomics features
      shape:
      firstorder:
      glcm:
        - 'Autocorrelation'
        - 'JointAverage'
        - 'ClusterProminence'
        - 'ClusterShade'
        - 'ClusterTendency'
        - 'Contrast'
        - 'Correlation'
        - 'DifferenceAverage'
        - 'DifferenceEntropy'
        - 'DifferenceVariance'
        - 'JointEnergy'
        - 'JointEntropy'
        - 'Imc1'
        - 'Imc2'
        - 'Idm'
        - 'Idmn'
        - 'Id'
        - 'Idn'
        - 'InverseVariance'
        - 'MaximumProbability'
        - 'SumEntropy'
        - 'SumSquares'
      glrlm:
      glszm:
      gldm:
```

© 2020 by the authors. Licensee MDPI, Basel, Switzerland. This article is an open access article distributed under the terms and conditions of the Creative Commons Attribution (CC BY) license (http://creativecommons.org/licenses/by/4.0/).
